# Nitazoxanide: A Drug Repositioning Compound with Potential Use in Chagas Disease in a Murine Model

**DOI:** 10.3390/ph16060826

**Published:** 2023-06-01

**Authors:** Minerva Arce-Fonseca, Rodolfo Andrés Gutiérrez-Ocejo, José Luis Rosales-Encina, Alberto Aranda-Fraustro, Juan José Cabrera-Mata, Olivia Rodríguez-Morales

**Affiliations:** 1 Laboratory of Molecular Immunology and Proteomics, Department of Molecular Biology, Instituto Nacional de Cardiología Ignacio Chávez, Juan Badiano No. 1, Col. Sección XVI, Tlalpan, Mexico City 14080, Mexico; mini_arce@yahoo.com.mx (M.A.-F.);; 2 Department of Infectomics and Molecular Pathogenesis, Centro de Investigación y de Estudios Avanzados del Instituto Politécnico Nacional (CINVESTAV-IPN), Av. Insituto Politécnico Nacional, Av. Instituto Politécnico Nacional No. 2508, Col. San Pedro Zacatenco, Gustavo A. Madero, Mexico City 07360, Mexico; 3 Department of Pathology, Instituto Nacional de Cardiología Ignacio Chávez, Juan Badiano No. 1, Col. Sección XVI, Tlalpan, Mexico City 14080, Mexico

**Keywords:** Chagas disease, *Trypanosoma cruzi*, drug repositioning, anti-*T. cruzi* agent, nitazoxanide, mouse model

## Abstract

Chagas disease (ChD), caused by *Trypanosoma cruzi*, is the most serious parasitosis in the western hemisphere. Benznidazole and nifurtimox, the only two trypanocidal drugs, are expensive, difficult to obtain, and have severe side effects. Nitazoxanide has shown to be effective against protozoa, bacteria, and viruses. This study aimed to evaluate the nitazoxanide efficacy against the Mexican *T. cruzi* Ninoa strain in mice. Infected animals were orally treated for 30 days with nitazoxanide (100 mg/kg) or benznidazole (10 mg/kg). The clinical, immunological, and histopathological conditions of the mice were evaluated. Nitazoxanide- or benznidazole-treated mice had longer survival and less parasitemia than those without treatment. Antibody production in the nitazoxanide-treated mice was of the IgG1-type and not of the IgG2-type as in the benznidazole-treated mice. Nitazoxanide-treated mice had significantly high IFN-γ levels compared to the other infected groups. Serious histological damage could be prevented with nitazoxanide treatment compared to without treatment. In conclusion, nitazoxanide decreased parasitemia levels, indirectly induced the production of IgG antibodies, and partially prevented histopathological damage; however, it did not show therapeutic superiority compared to benznidazole in any of the evaluated aspects. Therefore, the repositioning of nitazoxanide as an alternative treatment against ChD could be considered, since it did not trigger adverse effects that worsened the pathological condition of the infected mice.

## 1. Introduction

American trypanosomiasis, also known as Chagas disease (ChD), is caused by the protozoan parasite *Trypanosoma cruzi*, which is transmitted to humans and other mammals by blood-sucking hemipterans of the family Reduviidae. So far there are no vaccines to prevent ChD, and existing drugs are not 100% effective [1]. The World Health Organization reports that in the Americas alone, ChD has an annual incidence of 30,000 cases, 14,000 deaths per year, and 8000 cases of congenital infection [2]. ChD has been catalogued by the WHO within the list of neglected tropical diseases, which are characterized by being of parasitic, fungal, and bacterial infectious etiology that mainly affect populations living in poor socioeconomic conditions. It is considered a public health problem since it affects labor productivity, pregnancy, and child development [3]. ChD is endemic in 21 Latin American countries, and Mexico is the third country in prevalence with approximately 900,000 cases, only behind Brazil and Argentina [2,4]. 

The drugs used for the treatment of ChD are nifurtimox and benznidazole. Benznidazole is considered the first-line drug for its efficacy and for having a better toxicity profile than nifurtimox [5,6,7]; allergic dermatitis is the most frequent side effect, which is controlled with antihistamines [8]. Nifurtimox is considered in Mexico as the first-line drug because it is easier to obtain; however, it has less efficacy than benznidazole and more serious and potentially irreversible side effects, such as polyneuropathy [8]. For many years, it was believed that the administration of treatment offered no relief in chronic-stage patients; however, it has been proven that there is a benefit in patients who do not have advanced and irreversible cardiomyopathy when the drug is administered in the chronic stage of ChD [8].

Due to difficulties in obtaining the aforementioned drugs, their high cost, and mainly their serious side effects, people adhere poorly to treatment and eventually drop out. Therefore, several research groups have taken on the task of evaluating the efficacy of different compounds against *T. cruzi* as a better option for patients. As a consequence, drug repositioning is a useful tool to achieve better treatment outcomes [9]. Nitazoxanide is a possible candidate in the treatment of ChD because it is a drug that has a broad spectrum of action, low cost, acceptable tolerability, and its efficacy has already been reported against *T. cruzi* epimastigotes in vitro [10,11]. 

Nitazoxanide is an inhibitor of pyruvate/ferrodoxin oxidoreductases or PFORs, preventing the binding of pyruvate with thiamine pyrophosphate, which is a critical step in the anaerobic metabolism of protozoa [12]. The drug has an official indication for the treatment of diarrhea due to *Giardia lamblia* and *Cripstosporidium parvum* [13]. In addition to its official indications, nitazoxanide and its metabolite tizoxanide have been shown to be effective against numerous protozoa and helminths, including *Entamoeba histolytica*/*dispar*, *Blastocystis hominis*, *Trichuris trichuria*, *Ascaris lumbricoides,* and *Enterobius vermicularis* [14]. In terms of the effect on *T. cruzi*, nitazoxanide did not show encouraging results when used to treat ChD in a murine model infected with the *T. cruzi* Albarrada strain [15].

In this study, nitazoxanide was tested against the *T. cruzi* Ninoa strain as treatment in infected BALB/c mice, comparing its effectiveness in vivo with benznidazole.

## 2. Results

### 2.1. Parasitaemia, Body Condition and Survival

In order to evaluate the efficacy of nitazoxanide in controlling parasitemia and improving clinical presentation and survival, the number of parasites was quantified every other day from the tenth day after infection, clinical follow-up was performed, and daily mortality was recorded. Parasitemia occurred from day 13 post-infection in three groups (NTZ, infected and nitazoxanide-treated mice; BNZ, infected and benznidazole-treated mice; and W/O-T, infected mice without treatment). 

**Figure 1:**
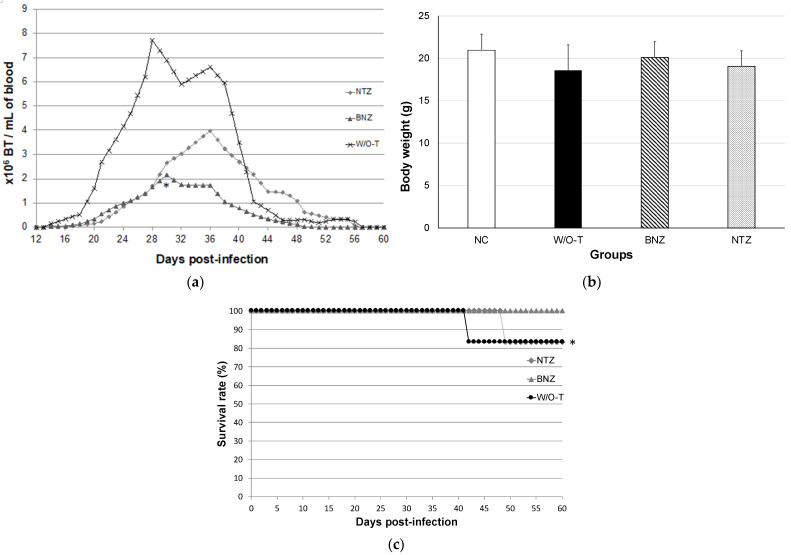
Parameters evaluating the effect of nitazoxanide treatment in a mouse model of experimental Chagas disease. (**a**) Parasitemia: using the Kruskal–Wallis test, the experimental groups were compared with the W/O-T control group, with statistical significance when * *p* ≤ 0.05. The blue line represents the period of time of administration of the treatments (from days 10 to 40 post-infection). (**b**) Body weight: using one-way ANOVA, body weight means of the experimental groups were compared with those of the NC group (negative control, healthy mice). (**c**) Survival rate: Kaplan–Meier curves demonstrating a significant difference when * *p* ≤ 0.05 between the W/O-T and NTZ groups compared to the BNZ group.

The BNZ group had patent parasitemia for 36 days (from day 13 to day 49), the W/O-T group had parasitemia for 43 days, and the NTZ group had parasitemia for 42 days. The BNZ group showed a peak of parasitemia on day 30, with 2.18 × 10^6^ blood trypomastigotes (BT)/mL, which was significantly lower than the W/O-T group; the NTZ group had a peak of parasitemia on day 36 post-infection, with 3.98 × 10^6^ BT/mL, which was almost half that of the untreated group (W/O-T group) but without being statistically significant from the W/O-T group; and finally, the W/O-T group presented two peaks of parasitemia at 27 and 36 days post-infection (dpi), with 7.7 × 10^6^ and 6.6 × 10^6^ BT/mL, respectively (Figure 1a). 

In the W/O-T group, severe signs of the acute stage of ChD were presented: curved spine, piloerection, and adynamia began to be visible from 29 dpi, whilst only piloerection was observed in the BNZ and NTZ groups from 36 dpi (data not shown). A body weight difference of 11.4% (18.58 g ± 3.01 g in the W/O-T group vs. 20.96 g ± 1.88 g in the negative control (NC) group), without a statistically significant difference (*p* = 0.632), was the largest recorded difference in body weight. Apparently, the benznidazole and nitazoxanide treatments ameliorated the clinical signs of acute-stage infection (Figure 1b).

The BNZ group had 100% survival, while in the NTZ and W/O-T groups, survival was 83.3%, with deaths registered on days 49 and 41 post-infection, respectively (Figure 1c). The data demonstrated that nitazoxanide was not as effective as benznidazole in reducing parasitemia and improving survival; however, mortality was prevented during the time that NTZ treatment administration was active, while in the W/O-T group, death was recorded earlier. In addition, the effects on health condition and weight loss were similar with both drugs.

### 2.2. Antibody Quantification

#### 2.2.1. Total IgG

**Figure 2:**
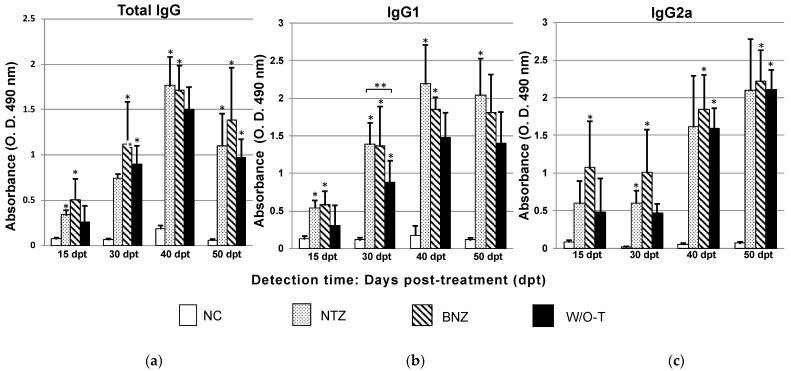
Serum antibody titers in a mouse model of Chagas disease after nitazoxanide treatment. (**a**) Total IgG, (**b**) IgG1 and (**c**) IgG2a. Using the Kruskal–Wallis test or one-way ANOVA, significant differences are shown with * *p* ≤ 0.05 when the infected groups were compared with the NC group (negative control, healthy mice), whose levels were below the cut-off value, and ** *p* ≤ 0.05 when the NTZ group was compared with the W/O-T group.

Levels of total immunoglobulins (IgG) in the NTZ (*p* = 0.037) and BNZ (*p* = 0.005) groups compared with those in the NC group after 15 days of treatment showed significant differences. After 30 days of treatment, a noticeable and significant increase in total IgG levels was observed in the mice from the W/O-T group (*p* = 0.007) as well as in the BNZ group, which persisted with a significant increase (*p* = 0.009). In contrast, at this time, no difference was found in the NTZ group with respect to the NC group. At 50 dpi, the NTZ group presented the highest amount of total IgG (*p* = 0.017) and, together with the BNZ group (*p* = 0.032), showed statistically significant differences with respect to the NC group. At 60 dpi, all of the infected groups, NTZ (*p* = 0.001), BNZ (*p* = 0.000), and W/O-T (*p* = 0.003), showed differences with respect to the NC group. This suggested that the effects of the nitazoxanide as well as benznidazole treatments were associated with the positive production of IgG-specific antibodies from the early phases of the acute stage of the infection (Figure 2a).

#### 2.2.2. IgG1

The IgG1 levels in the NTZ (*p* = 0.005) and BNZ (*p* = 0.005) groups 15 days after treatment showed significant differences when compared to the levels found in the NC group.

All three infected groups showed differences from the NC group on day 30 of treatment, and there was an intergroup difference (*p* = 0.049) between the NTZ and W/O-T groups. At 40 dpt, the BNZ (*p* = 0.040) and NTZ (*p* = 0.004) groups differed from the NC group. Finally, at 50 dpt, the group treated with nitazoxanide was the only one that showed a difference (*p* = 0.003) in the production of the IgG1 subclass (Figure 2b). This suggested that while the infected mice of the W/O-T and BNZ groups showed declining IgG1 levels, probably to switch to the IgG2a subclass, nitazoxanide treatment caused persistent high IgG1 subclass levels, as a likely consequence of sustained inflammation.

#### 2.2.3. IgG2a

Only the BNZ group presented a significant difference in IgG2a levels (*p* = 0.043) at 15 days of treatment. The NTZ (*p* = 0.020) and BNZ (*p* = 0.003) groups differed at 30 days of treatment. At 40 days, only the BNZ *(p* = 0.026) and W/O-T (*p* = 0.050) groups showed a difference in IgG2a levels, which continued in both groups at 50 dpt (Figure 2c). It could be concluded that the effect of nitazoxanide was not associated with an increase in IgG2a production; therefore, this drug was not able to shift the subclass of IgG-specific antibodies from IgG1 to IgG2a at the end of the acute stage of infection. 

### 2.3. Serum Cytokine Levels 

**Figure 3:**
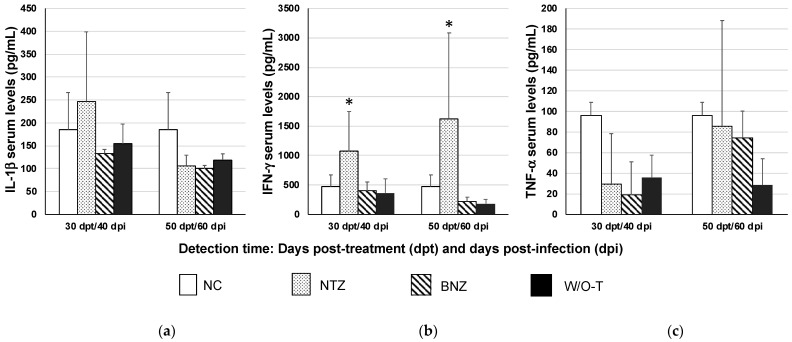
Quantification of serum cytokines in a mouse model of Chagas disease after nitazoxanide treatment. The values represent the group mean ± S.D. for detection of IL-1β (**a**), IFN-γ (**b**), and TNF-α (**c**). Using the Kruskal–Wallis test or one-way ANOVA, significant differences are shown with * *p* ≤ 0.05 when comparing the infected groups with the NC group (negative control, healthy mice).

Interferon-gamma (IFN-γ) was the only serum cytokine that showed a significant increase in the NTZ group in both stages of infection: acute (40 dpi and 30 days post-treatment [dpt]) and asymptomatic chronic (60 dpi/50 dpt), unlike tumor necrosis factor-alpha (TNF-α) and interleukin-1 beta (IL-1β), the serum levels of which did not differ from the baseline level determined in the NC group mice (Figure 3).

### 2.4. Organ Indices to Determine Cardiomegaly, Esplenomegaly and Lymphadenopathy 

**Figure 4:**
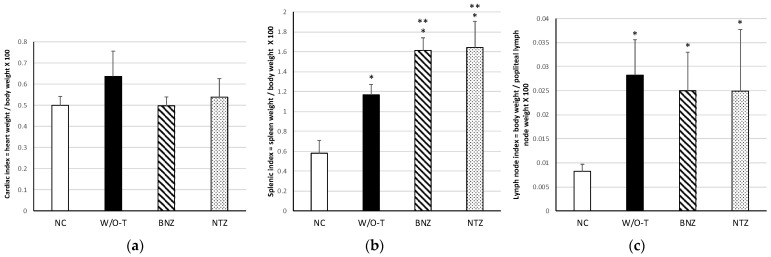
Organ indices in a mouse model of Chagas disease after nitazoxanide treatment. Cardiac (**a**), splenic (**b**), and lymph node (**c**) indices were evaluated at 60 dpi. Using the Kruskal–Wallis test, significant differences are shown with * *p* ≤ 0.05 when the infected groups were compared with the NC group (negative control, healthy mice), and ** *p* ≤ 0.05 when the infected groups were compared with the W/O-T group.

Although no statistically significant difference was found between the infected and healthy mice, the data showed a higher cardiac index in the mice that did not receive treatment and the lowest value was in the benznidazole-treated group, as expected (Figure 4a). 

All groups of infected mice showed a statistically significant difference in the splenic index with respect to the healthy non-infected group (NC): NTZ (*p* = 0.000), BNZ (*p* = 0.000), and W/O-T (*p* = 0.015) groups; therefore, it was possible to affirm the existence of splenomegaly, which is a characteristic sign of ChD. In addition, differences were observed between the NTZ (*p* = 0.018) and BNZ (*p* = 0.047) groups when they were compared only with the W/O-T group, which demonstrated that splenomegaly was even greater in those animals who received treatment (Figure 4b).

Lymphadenopathy was found in all of the infected groups, NTZ (*p* = 0.028), BNZ (*p* = 0.042), and W/O-T (*p* = 0.005), when compared with the healthy group. This was probably due to a reaction caused by the persistence of the parasite in nearby myocytes. The infected groups did not show differences among them (Figure 4c).

### 2.5. Histological Findings and Severity of Inflammation

**Figure 5:**
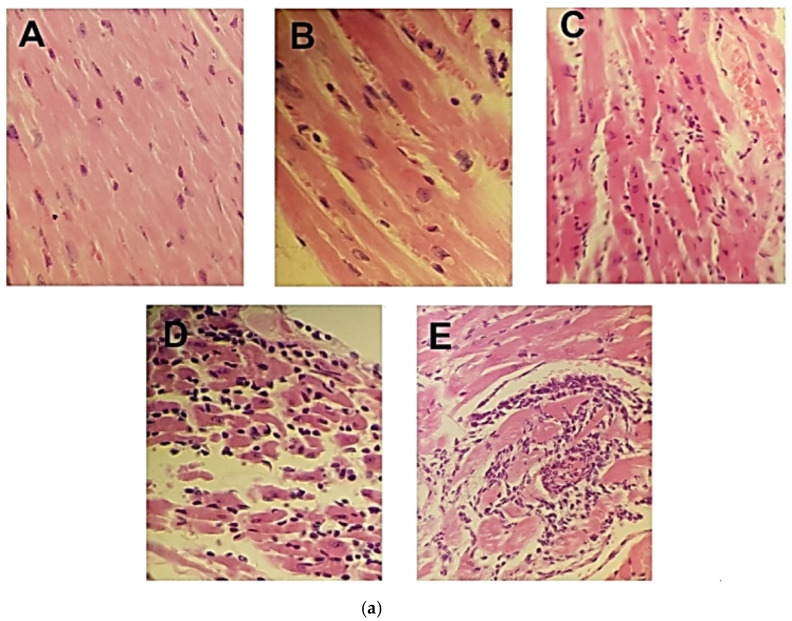

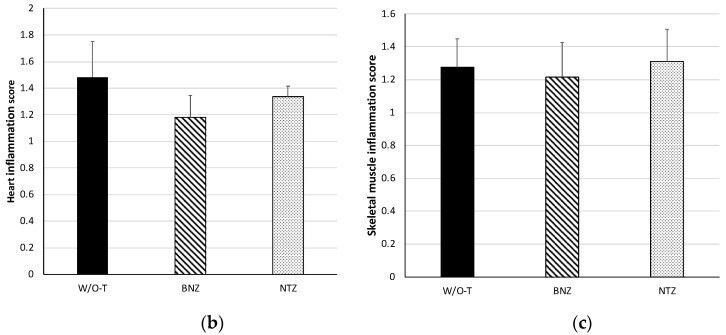
Histological damage and degree of inflammation of cardiac and skeletal muscle in a mouse model of Chagas disease after nitazoxanide treatment. (**a**) Microscopic damage of representative micrographs of cardiac tissue: NC group (negative control, healthy mice) (**A**); BNZ group with a score of 1 (**B**) and a score of 2 (**C**); NTZ group with a score of 3 (**D**), and W/O-T with a score of 4 (**E**) are shown. (**b**) Heart and (**c**) skeletal muscle inflammation scores. The score was obtained as previously described [16]. Using the Kruskal–Wallis test, no significant differences were observed when comparing the NTZ and BNZ groups with the W/O-T group.

**Figure 6:**
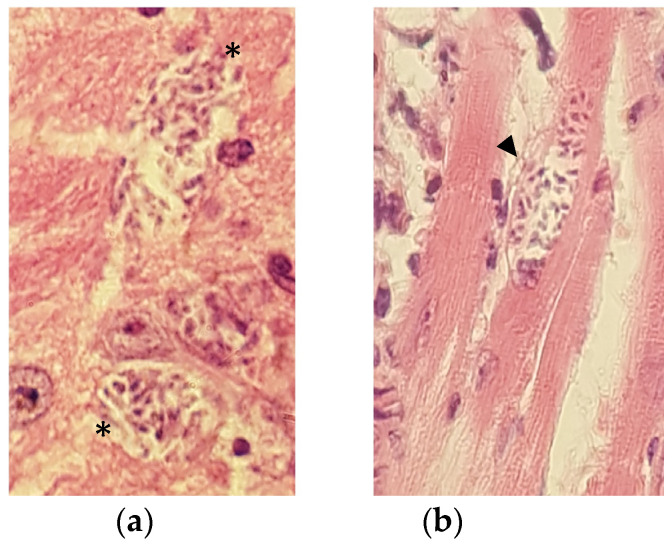
Amastigotes nests in brain and heart of a mouse model of Chagas disease without treatment. (**a**) Parasites (asterisks) in the center of midbrain area with vacuolization and ischemia (not photographed). (**b**) Amastigotes nest (arrowhead) in myocardium section from an untreated mouse. Hematoxylin and eosin stain, 40X objective.

Mononuclear infiltrate was found in all mice in the infected groups, located in the myocardium (predominantly sub-epicardial) (Figure 5a) and skeletal muscle. Although there was no statistically significant difference among the groups, higher inflammation scores in heart and skeletal muscle were obtained in the group without treatment (Figure 5b,c), in addition to the presence of nests of amastigotes in the myocardium (Figure 6a) and in the midbrain (Figure 6b), follicular hyperplasia of the spleen, and sinusoidal dilatation (data not shown). No alterations were found in the other organs examined.

## 3. Discussion

In the present study, nitazoxanide treatment against experimental ChD was evaluated in vivo based on its effects on parasitemia, survival, body condition, immune response, and macroscopic and microscopic tissue damage in BALB/c mice in the acute stage of *T. cruzi* infection. 

Animals treated with nitazoxanide had greater parasitemia than those that were treated with benznidazole, as reported by others [15], who observed that mice that received nitazoxanide showed greater parasitemia than those that were treated with nifurtimox (drug equivalent to benznidazole), as well as longer duration of parasitemia, even than the group without treatment. However, these data were not consistent with the present study’s findings. It should be noted that the nitazoxanide treatment scheme used by those authors had variations in dose and administration time, since they used 100 and 1000 mg/kg for 20 days, starting on the first day of parasitemia (3 dpi); in the present study, 100 mg/kg was used at the beginning of parasitemia (10 dpi) for 30 days. These small variations were enough to observe a favorable result. The NTZ group showed a lower level of circulating parasites than the group that did not receive treatment, demonstrating an effect similar to that of benznidazole with trypanocidal activity against the *T. cruzi* Ninoa strain, unlike the data reported by those authors who observed that their group treated with nitazoxanide showed higher parasitemia than the untreated animals [15].

In addition to the nitazoxanide therapeutic scheme, the discrepancy between results could be explained by the *T. cruzi* strain and the size of the inoculum used to infect the experimental mice in each of the studies. For example, Valle-Reyes et al. used 1 × 10^5^ BT of the TPAP/MX/2002/Albarrada strain and male BALB/c mice [15], whereas in the present study, the experimental infection in female BALB/c mice was with 150 BT of the MHOM/MX/1994/Ninoa strain. There are factors inherent to the parasite that cause differential virulence and pathogenicity parameters among lineages or DTU variants of *T. cruzi* strains [17,18,19,20,21]. 

In this study, 48% reduction in parasitemia was obtained with nitazoxanide, which was comparable to 42% reported by other authors who used clomipramine (a tricyclic antidepressant) also in a murine model [22]. In comparison, the use of voriconazole (second-generation triazole antimycotic) achieved 80% reduction [23], and VNI (sterol 14α-demethylase (CYP51) inhibitor) and its derivative VFV achieved reductions in parasitemia of 99.7% and 91% to 100%, respectively [24]. These rates of reduction in parasitemia demonstrate wide ranges of effectiveness, which is related to the *T. cruzi* strain used in the infection as well as the therapeutic target of the drug evaluated.

As expected, the survival of the group treated with benznidazole was 100%; however, it was 83% in the NTZ group, a rate much higher than that reported by Valle-Reyes et al. (2017), who observed a survival of only 20% [15]. It is known that the Albarrada strain has a polyclonal structure, which may be responsible for its differential behavior of damage and infection in different hosts [25]. This argument could explain why this strain exacerbated its virulence in nitazoxanide-treated mice in the study by Valle-Reyes et al. [15]. Despite both strains (Albarrada and Ninoa) being Mexican and belonging to discrete typing unit I (DTU I), they have different behaviors in terms of parasitemia and survival in mice, parameters that are associated with inherent factors of the parasite such as virulence and pathogenicity. In other studies in which the drug repositioning strategy has been used for the treatment of infection with *T. cruzi* strains Y or Tulahuen in a murine model, it was observed that survival depended on the specificity of the mechanism of action of the drug used. For example, mice treated with clomipramine (an antidepressant drug), voriconazole (antimycotic drug), and VFV and VNI drugs (aimed at inhibiting enzymes involved in parasite metabolism) had 50%, 83%, and 100% survival rates, respectively [22,23,24].

The nitazoxanide-treated group presented a lower average body weight than all of the other groups, most likely due to some side effects of the drug (anorexia, diarrhea, and nausea) that probably caused a lower feed intake and weight loss, or a direct catabolic effect of the drug, since some studies showed that although it can be well tolerated due to its good safety profile, it may be associated of adverse reactions, especially when prolonged and increased oral doses are administered [26,27,28]. 

The control of parasitemia by benznidazole and to a lesser extent by nitazoxanide was consistent with the findings for IgG antibodies and cytokines in acute and early chronic stages of ChD. In both groups, IgG antibody production could be observed from 15 dpt (25 dpi), which agreed in part with some results reported by Espinoza et al., who found immunoglobulins in the acute phase of the infection [29]. All of the infected groups showed significant levels of total IgG at 60 dpi in an asymptomatic chronic phase of the disease.

Negative seroconversion was not observed in the infected and treated groups, most likely due to the short evaluation time (60 dpi), since in other studies it has been reported that antibodies against *T. cruzi* disappeared in animals treated with benznidazole at 3–12 mpi [30,31].

There was a statistically significant increase in IgG1 production in the NTZ group but not in the BNZ group at day 60 post-infection. Therefore, it is suggested that nitazoxanide treatment preferentially promoted a shift to a Th2 immune response. In the case of benznidazole, the result was slightly different, since the prevalent isotype was IgG2a with a predominance of a Th1-type immune response, which could be related to a reduction in parasitemia [32]. In addition, antibody production influences inflammatory cell infiltration in tissues; therefore, it has been observed that the inflammatory process, mainly in cardiac tissue, is directly related to the levels of IgG [33]. This finding agrees with our work, in which nitazoxanide-treated mice were found to have slightly higher organ indices and inflammation scores than benznidazole-treated mice, with the latter showing lower IgG1 levels and higher IgG2 levels, indicating better infection control through a balanced Th1/Th2 immune response as the disease progressed.

In the NTZ group, IFN-γ was the unique cytokine observed in significantly high levels compared to other two infected groups (BNZ and W-O/T). This cytokine has been linked to host defense in the acute phase [34]. On the other hand, studies have also reported that the cytokine profile showed decreased IL-1β, IL-10, and TNF-α levels after benznidazole treatment [35]. This is consistent with the decreased IL-1β and TNF-α levels observed at 60 and 30 dpi, respectively, with both benznidazole and nitazoxanide treatments, although without a significant difference. In the NTZ group at 40 and 60 dpi, there were high concentrations of IFN-γ, which were not associated with the other results obtained such as moderate control of parasitemia and slight histological damage.

It has been reported that *T. cruzi* Ninoa strain induces splenomegaly [29]. It was shown that all of the infected groups had splenomegaly; the treated groups (NTZ and BNZ) showed a higher splenic index than the W/O-T group, and of these, the BNZ group presented the highest index. In turn, the BNZ group registered the shortest duration of parasitemia and the highest survival rate. Therefore, it can be deduced that the presence of splenomegaly resulted in a greater immune response and better infection control, probably due to lymphocyte proliferation with positive activity against *T. cruzi* infection leading to the abundance of a kind of lymphocyte with a cytotoxic phenotype in the spleen of infected mice [36,37]. 

The three infected groups presented statistically significant growth of lymph nodes; the group without treatment had the highest index and also showed microscopic changes such as hepatic sinusoidal dilatation, which was probably associated with giant lymph node hyperplasia, as has been reported in ChD and other pathologies [38,39,40].

Although cardiomegaly was not determined due to the absence of a statistically significant difference in the cardiac index between the infected and healthy groups, a higher cardiac index suggestive of cardiomegaly was observed in the group that did not receive treatment, unlike the BNZ group, which had a cardiac index similar to that of the NC group. The cardiac indices of the nitazoxanide-treated animals were at a midpoint between those of the BNZ and W-O/T groups, therefore affirming that the progression to cardiomegaly was partially decreased in comparison with not receiving treatment. These results were consistent with the important pathological outcome of chronic chagasic cardiomyopathy, since multiple factors such as severe myocarditis and fibrosis cause a hemodynamic overload of the heart, which represents a compensatory mechanism that leads to myocardial hypertrophy [41]. On the other hand, it has been reported that nitazoxanide induces myocardial injury by activating the oxidative stress response [42]. This may be the reason why the NTZ group’s cardiac index did not have low values similar to those of the NC group, or at least similar to those of the BNZ group. Therefore, there is a paradox in the therapeutic use of nitazoxanide against ChD, since possible heart damage as a side effect must be evaluated with greater precision.

One of the limitations of the present study was that only three types of cytokines of the Th1 profile of the immune response were analyzed; therefore, a greater number of these would provide more complete information on the type of immune response triggered. Another limitation was that no adverse effects other than body weight were measured, and although these are already known, it would be useful to ascertain if these can synergize with the parasitic infection. As a perspective, it would be interesting to evaluate the parameters in a more advanced chronic stage of ChD, in which it would be possible to analyze the drug’s effect in the long term. On the other hand, nitazoxanide could be used in combination with benznidazole to improve its trypanocidal effectiveness, as noted by several authors when combining different drugs with benznidazole [22,23,31,43,44,45,46,47].

## 4. Materials and Methods

### 4.1. Model Animal

The study was carried out with 72 female, 6–8-week-old, BALB/c mice obtained from the Laboratory Animal Experimentation and Production Unit (UPEAL) of CINVESTAV-IPN, Mexico. The animals were separated into groups in cages and maintained in a macroenvironment with a 12 h/12 h light-dark cycle, 20–22 °C temperature, and 40–50% relative humidity. Water and food (Purina Lab Diet Formula 5001^®^, Silao, Gto., Mexico) were available ad libitum. Mice were divided into four groups, three of which were infected, with one treated with nitazoxanide (*n* = 12, NTZ), another with benznidazole (*n* = 8, BNZ), one without treatment (*n* = 12, W/O-T), and a fourth group was not infected nor was it given any treatment, thus representing the negative control (*n* = 4, NC). The experiment was performed in duplicate. 

The Bioethics Committee of the Instituto Nacional de Cardiología Ignacio Chávez is ruled according to the International Guiding Principles for Biomedical Research involving Animals and the Norma Oficial Mexicana (NOM-0062-ZOO-1999) Technical Specifications for the Care and Use of Laboratory Animals [48]. This committee was responsible for reviewing and approving this research.

### 4.2. Infection, Parasitaemia and Survival

Mice were inoculated intraperitoneally with 150 BT of the *T. cruzi* Mexican Ninoa strain (MHOM/MX/199/Ninoa), a strain belonging to discrete typing unit I (DTU I), which is the most widely distributed strain throughout the Americas and causes myotropism [49,50], in 200 µL of saline solution (SS) (0.9% NaCl) using a 1 mL syringe and a 27 G × 13 mm needle (Becton- Dickinson ^®^, Mexico City, Mexico). From day 10, a 1:50 dilution was made adding 10 µL of peripheral blood extracted through the caudal vein to 490 µL of SS, and parasitemia was quantified every other day in a Neubauer counting chamber by light microscopy until parasites were no longer observed. Survival was monitored daily.

### 4.3. Nitazoxanide and Benznidazole Treatment

Drug suspensions were made using nitazoxanide (Daxon^®^ 500 mg tablets, Siegfried-Rhein Laboratory, Mexico City, Mexico) and benznidazole (LaFepe^®^ 100 mg tablets, Laboratório Farmaceutico do Estado de Pernambuco, Recife, Brazil) in Tween 5% and SS. Nitazoxanide and benznidazole were administered at doses of 100 and 10 mg/kg of body weight/24 h, respectively, both in a volume of 500 µL of the previously described suspension, by the orogastric route using a 1 mL syringe and a straight feeding cannula of stainless-steel No. 18 (ScientMex^®^, Monterrey, NL, Mexico). Treatments were administered from day 10 post-infection, lasting 30 days.

### 4.4. Serum Sample Collection

About 250–300 µL of peripheral blood was collected through the caudal vein before infection, at 15 and 30 days of treatment, and 60 days after infection. Blood was placed in 1.5 mL microcentrifuge tubes; once the clot was retracted, the sample was centrifuged at 3500 rpm for 15 min at 4 °C in a refrigerated micro-centrifuge (RMC-14 model, Sorvall^®^/DuPont^®^, Lynn, MA, USA). Finally, the serum was separated, and aliquots were frozen.

### 4.5. Antibody Quantification

Determination of immunoglobulin G and its subclasses was performed by enzyme-linked immunosorbent assay (ELISA) using the sera obtained for each group, as previously described [16]. Anti-mouse peroxidase-conjugated antibodies (Novus Biologicals, Littleton, CO, USA) and anti-mouse IgG, IgG1, and IgG2a were used. The cut-off point (mean ± standard deviation, SD) was established according to the values obtained for the samples from healthy mice (NC group). 

### 4.6. Serum Cytokine Quantification

Commercial ELISA kits (Enzo Life Sciences, Inc.^®^, Farmingdale, NY, USA and R&D Systems, Inc.^®^, Minneapolis, MN, USA) were used to determine levels of some proinflammatory cytokines (TNF-α, IFN-γ, and IL-1β,) in the mouse sera at 40 and 60 days after infection, according to the manufacturer’s instructions and as described previously [16].

### 4.7. Determination of Visceral Megas

The mice were weighed before euthanasia; subsequently, the heart, spleen, and both popliteal lymph nodes were collected and weighed. Heart, splenic, and lymph node indices were obtained using the formula: Organ index = organ weight/body weight × 100. The results were compared with the indices of infected mice without treatment (W/O-T group) and those of non-infected healthy mice (NC group).

### 4.8. Histology

The mice were euthanized on day 60 post-infection and the following organs were collected: the heart, spleen, esophagus, large intestine, small intestine, brain, skeletal muscle, and peripheral lymph nodes (poplitei). After histological processing, the tissue sections were stained with hematoxylin and eosin for analysis, as previously described [16].

A score of 1 to 4 was given to classify microscopic damage as follows: 1, one focus of inflammation at 400× magnification; 2, more than one inflammatory focus; 3, coalescing foci or generalized inflammation, with conservation of normal structure and minimal cell necrosis; and 4, non-localized inflammation, tissue necrosis, fluid accumulation in the interstitial compartment, and alteration of tissue structure [51]. The data obtained from the score were corrected as previously described [52].

### 4.9. Statistical Analysis

The whole data were examined using IBM^®^ SPSS software version 20.0.0 (Armonk, NY, USA). The Shapiro–Wilk test was applied to verify that the data had a normal distribution; depending on the distribution of the data, one-way ANOVA (Tukey’s post hoc test) or the Kruskal–Wallis test was performed, and the difference was considered significant when *p* ≤ 0.05.

## 5. Conclusions

Nitazoxanide decreased parasitemia levels, indirectly induced the production of IgG antibodies, and partially prevented histopathological damage in the infected mice; however, it did not show therapeutic superiority compared to benznidazole in any of the evaluated aspects. Therefore, nitazoxanide administered to mice in the acute stage of infection with *T. cruzi* was moderately effective against ChD, so it can be considered as an alternative therapeutic candidate to benznidazole since it did not trigger negative effects that worsened the pathological condition of the infected mice.

## Data Availability

Data is contained within the article.
